# Parkinsonian motor impairment predicts personality domains related to genetic risk and treatment outcomes in schizophrenia

**DOI:** 10.1038/npjschz.2016.36

**Published:** 2017-01-11

**Authors:** Juan L Molina, María Calvó, Eduardo Padilla, Mara Balda, Gabriela González Alemán, Néstor V Florenzano, Gonzalo Guerrero, Danielle Kamis, Beatriz Molina Rangeon, Mercedes Bourdieu, Sergio A Strejilevich, Horacio A Conesa, Javier I Escobar, Igor Zwir, C Robert Cloninger, Gabriel A de Erausquin

**Affiliations:** 1 Department of Psychiatry and Behavioral Neurosciences, Morsani College of Medicine, University of South Florida, Tampa, FL, USA; 2 Fundación de Lucha contra los Trastornos Neurológicos y Psiquiátricos en Minorías, FULTRA, Buenos Aires, Argentina; 3 Hospital Neuropsiquiátrico Dr Néstor Sequeiros, San Salvador de Jujuy, Argentina; 4 Unidad de Neurociencias, Dr J.J. Naón, Facultad de Medicina, Universidad de Buenos Aires, Buenos Aires, Argentina; 5 Rutgers University-Robert Wood Johnson Medical School, New Brunswick, NJ, USA; 6 Departments of Psychiatry and Genetics, Washington University School of Medicine, St Louis, MO, USA; 7 Division of Neurosciences and Department of Psychiatry and Neurology, UTRGV School of Medicine, Harlingen, TX, USA

## Abstract

Identifying endophenotypes of schizophrenia is of critical importance and has profound implications on clinical practice. Here we propose an innovative approach to clarify the mechanims through which temperament and character deviance relates to risk for schizophrenia and predict long-term treatment outcomes. We recruited 61 antipsychotic naïve subjects with chronic schizophrenia, 99 unaffected relatives, and 68 healthy controls from rural communities in the Central Andes. Diagnosis was ascertained with the Schedules of Clinical Assessment in Neuropsychiatry; parkinsonian motor impairment was measured with the Unified Parkinson’s Disease Rating Scale; mesencephalic parenchyma was evaluated with transcranial ultrasound; and personality traits were assessed using the Temperament and Character Inventory. Ten-year outcome data was available for ~40% of the index cases. Patients with schizophrenia had higher harm avoidance and self-transcendence (ST), and lower reward dependence (RD), cooperativeness (CO), and self-directedness (SD). Unaffected relatives had higher ST and lower CO and SD. Parkinsonism reliably predicted RD, CO, and SD after correcting for age and sex. The average duration of untreated psychosis (DUP) was over 5 years. Further, SD was anticorrelated with DUP and antipsychotic dosing at follow-up. Baseline DUP was related to antipsychotic dose-years. Further, ‘explosive/borderline’, ‘methodical/obsessive’, and ‘disorganized/schizotypal’ personality profiles were associated with increased risk of schizophrenia. Parkinsonism predicts core personality features and treatment outcomes in schizophrenia. Our study suggests that RD, CO, and SD are endophenotypes of the disease that may, in part, be mediated by dopaminergic function. Further, SD is an important determinant of treatment course and outcome.

## Introduction

Schizophrenia is a heritable disorder that causes great psychosocial and interpersonal impairment. Advances in the care and treatment of severe mental illness in indigenous Latin American populations are lacking.^1^ In the case of the indigenous communities in the Central Andes, the problem is further compounded by limited road availability and mountainous terrain, leading to little exposure to western medical practices. Kechwa language is the most prevalent in the region, with more than 10 million speakers, and includes terms to describe severe depression (llaqui onqoy), mania (taqui onqoy), and non-affective psychosis not caused by herbs or alcohol (utiqay)*;* the latter approximating schizophrenia.^2^ Such terminology correlates with the ability of traditional local healers to diagnose cases of mental illness.^3^ Taking advantage of this unique combination of a population largely unexposed to medications but able to relate to the concept of psychosis, we assessed the role of parkinsonism in chronic untreated schizophrenia, and its relationship to behavioral, cognitive and imaging endophenotypes of risk in unaffected first-degree relatives.^4–7^

Kraepelin described prominent motor abnormalities (lack of facial expression, rigidity, slowness, and paucity of movement) along with the better-known psychiatric phenomenology.^8^ The introduction of neuroleptics shifted all emphasis on the presence of such abnormalities to medication side effects, all but ignoring their contribution to the disease process. The discovery of neuromotor abnormalities in pre-schizophrenic children renewed the issue,^9,10^ leading to confirmations of parkinsonian motor abnormalities in first-episode psychosis^11–14^ and in treatment naive chronic schizophrenia.^5,15–19^ Yet, to the best of our knowledge the relationship of parkinsonian motor impairment as mediators of personality traits and clinical outcomes in antipsychotic naïve patients with schizophrenia and in their unaffected relatives has not been explored.

Indeed, in Parkinson's disease, a relationship has been clearly established between deviant personality traits and basal ganglia dysfunction,^20,21^ and we recently showed that parkinsonian motor impairment and substantia nigra hyperechogenicity, a biomarker of Parkinson disease, are present in neuroleptic naive subjects with chronic schizophrenia and in their unaffected first-degree relatives.^5^

Therefore, we hypothesized that parkinsonian motor impairment and hyperechogenicity of the substantia nigra would predict personality traits also known to correlate with decreased dopaminergic function, the latter acting as mediators to longer duration of untreated psychosis and poorer clinical outcomes.

Personality structures are temporally stable across cultural contexts,^22^ and discrete configurations of personality are heritable and reflect risk for schizophrenia.^4,23,24^ Thus, the psychobiological model of personality provides a heuristic explanation for the relationship of traits associated with risk of disease, pathophysiology, and clinical outcome^25–27^ including the role of cortical and subcortical structures on individual differences in personality traits.^28–31^

Personality profiles of subjects with schizophrenia and their unaffected relatives have been studied using the Temperament and Character Inventory (TCI) in a variety of cultural environments and languages around the world, finding a disease-state related pattern consisting of higher harm avoidance and ST with lower reward dependence (RD), persistence, cooperativeness, and self-directedness (SD).^4,23,32–35^ These personality traits have been related to symptom profile^36,37^ and psychosocial function^27,38,39^ in patients with chronic schizophrenia. In unaffected relatives, deviance in character dimensions is consistent with schizotypal traits,^4,23,40^ but with some variation across samples.^24,32,41^

We sought to further elucidate the role of personality structures in the risk architecture of schizophrenia by including unaffected relatives and healthy controls (matched to the index subjects) in our sample. Specificaly, we proposed to test if an endophenotype associated with parkinsonism and midbrain hyperechogenicity predicts deviance on personality traits. We proposed to test the role of personality deviance as a mediator between parkinsonism and poor clinical outcomes over the long-term course of the illness.

## Results

### Description of the sample

We studied 61 subjects with schizophrenia, 99 unaffected relatives (59 siblings and 40 parents), and 68 healthy controls (Table 1). Average age (mean±s.d.) was 28±11 for affected subjects, 40±15 for unaffected relatives, and 34±14 for healthy controls. There were significantly more males (60%) among the index subjects, and more females (64%) among the unaffected relatives. Years of education were, on average, 10 for subjects with schizophrenia, 10 for relatives, and 11 for healthy controls. None of the affected subjects was married, and all were unemployed. Mean DUP was >5 years.

### Temperament and Character Inventory scores for individual scales differ between risk groups

*Temperament scales*. Subjects with schizophrenia had higher harm avoidance (HA) (F=3.5, *P*=0.01) than both unaffected relatives and controls. Subjects also had lower reward dependence (RD) (F=11.2, *P*<0.001) than both unaffected relatives and controls.

**Figure 1:**
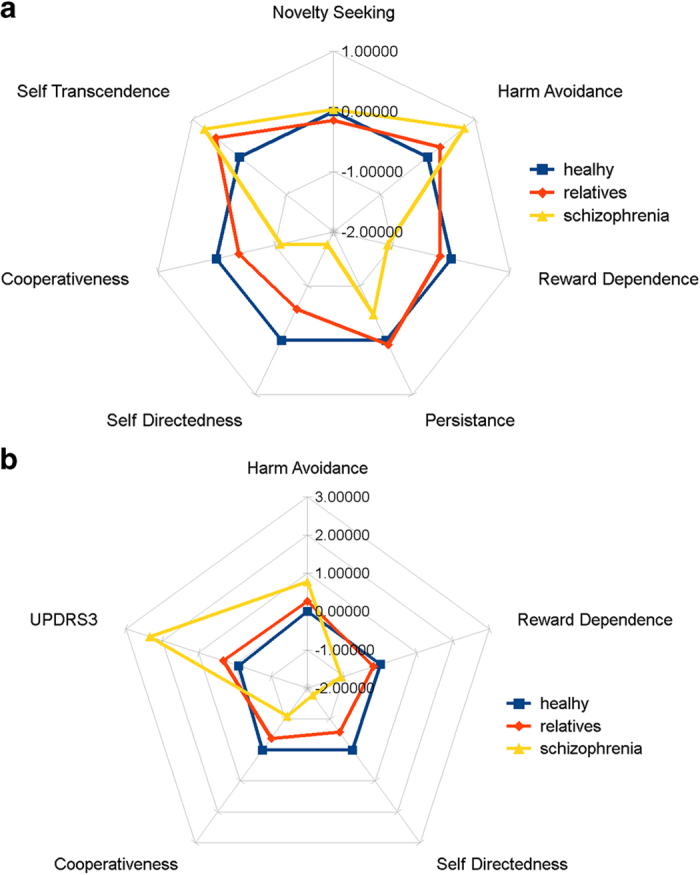
Net charts showing *Z*-scores (0=mean for healthy controls, 1=SD of the mean) for TCI domains (**a**) and parkinsonism and dopamine-modulated traits (**b**). Deviance on individual variables for patients with schizophrenia (yellow triangle) and unaffected relatives (red diamonds) were normalized relative to controls (blue squares).

*Character scales*. Affected subjects and unaffected relatives had significantly higher ST (F=3.4 *P*=0.011) than controls, however, there were no differences between patients and unaffected relatives. Both CO (F=8.8, *P*<0.001) and SD (F=20.0, *P*<0.001) follow a graded distribution where subjects scored lowest, unaffected relatives were intermediate (but significantly different than patients and controls), with controls scoring the highest. See Figure 1a and Table 1 for summary profiles of TCI domains.

### Parkinsonism and substantia nigra echogenicity differ between risk groups

UPDRS3 scores followed a graded distribution with subjects with schizophrenia being worst, and their unaffected relatives significantly worse than healthy controls (See Table 1; F=41.7, *P*<0.001). On the other hand, echogenicity of the right (but not left) substantia nigra was significantly increased in the affected subjects compared with both unaffected relatives and controls, (Table 1; F=7.3, *P*<0.001), as would be expected for a marker of genetic risk.

### Parkinsonism and SN echogenicity are linear predictors of scores for individual TCI scales

**Figure 2:**
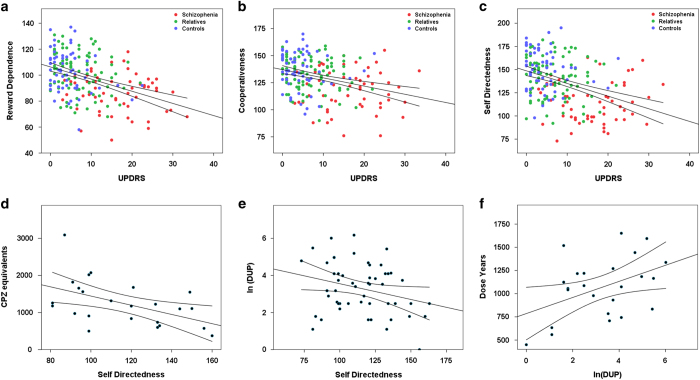
Parkinsonism predicts core personality traits in schizophrenia and the relationship of self-directedness to course of illness and outcomes. (**a**–**c**) Linear regression between UPDRS3 scores and reward dependence (**a**, R^2^=0.265, *P*<0.001), cooperativeness (**b**, R2=0.108, *P*=0.001), and self-directedness (**c**, R^2^=0.183, *P*<0.001). 95% Intervals of confidence are also shown. R^2^-values and significance are reported after correcting for age, sex, and multiple comparisons. In all three cases, severity of motor impairment predicts lower expression of the trait. Unaffected relatives (green dots) consistently appear between patients (red dots) and healthy controls (blue dots). Panels (**d**,**e**). display regression lines using self-directedness as predictors of duration of untreated psychosis (**e**, R^2^=−0.075, *P*=0.046) and average neuroleptic dose in chlorpromazine equivalents (**d**, R^2^=−0.251, *P*=0.013). (**f**) The predicted relationship between duration of untreated psychosis and cumulative neuroleptic dose (**f**, R^2^=0.193, *P*=0.032).

Figure 1b summarizes the differences in parameters associated with dopaminergic brain function, between subjects with chronic untreated schizophrenia, their unaffected first-degree relatives and healthy controls on a z-space (mean=0, SD=1). Linear regressions for individual TCI scales using UPDRS and SN echogenicity as predictors and age and sex as covariates in the model revealed significant relationships for HA, RD, PS, CO, and SD (see Table 2). However, after correcting for multiple comparisons (*P*=0.008) only RD, SD, and CO remained significant (see Figures 2a–c, Table 2).

### Discriminant Function Analysis using parkinsonism, midbrain echogenicity, and personality scores as predictors of schizophrenia risk

To address the classification ability of an intermediate phenotype reflecting dopaminergic deficits, we carried out a canonical discriminant analysis including those variables that were significantly different between groups, namely the TCI scales, parkinsonism, and right SN echogenic area. Two discriminant functions were significant and accounted for 98.5% and 1.5% of the total variance respectively, with canonical correlation coefficients of 0.71 (*χ*^2^=122, *P*<0.001) and 0.12 (χ^2^=2.6, *P*>0.05). The DF1 structure matrix received the largest variance contributions from UPDRS3, SD, RD, CO, right SN echogenic area, and HA, while DF2 received variance contributions from ST and PS (see Supplementary Table 1). Predictions of group membership (affected, unaffected relative, healthy control) based on DF scores were correct for 65.7% of all the cases included, regardless of risk status, with 80% of affected subjects and 70% of healthy controls being correctly classified.

Most classification errors were due to unaffected relatives, most likely because of their intermediate expression of disease-related phenotypes. Therefore, we analyzed the distribution of variables in unaffected relatives grouped by predicted category membership assignment (i.e., misclassified as ‘schizophrenia’ against all other unaffected relatives) in order to assess the contribution of specific variables to assignment errors, which could serve as a proxy markers of genetic risk. A multivariate analysis of variance (MANOVA) revealed a significant group effect (for predicted membership) (F=6.6, observed power=1.0, *P*<0.001); unaffected relatives misclassified as subjects with schizophrenia had significantly greater parkinsonism (F=14.6, *P*<0.001), and lower RD (Figure 2b, F=6.1, *P*=0.016), CO (F=6.8, *P*=0.011), and SD (F=30.1, *P*<0.001). There were no significant differences in right substantia nigra, PS, ST between subgroups of unaffected relatives.

### Specific TCI scales are predictive of DUP in unmedicated chronic schizophrenia

To assess the relationship between personality domains and predictors of severity of illness and disability, we derived exploratory bivariate correlations between TCI domains and DUP. We found a significant negative correlation between SD and DUP (Figure 2e. R^2^=0.075, *P*=0.046, two-tailed). No other factors reached statistical significance.

### Specific TCI scales and DUP are predictive of medication use at long-term follow-up

In a subgroup analysis where clinical follow-up data were available (*n*=24), we sought to explore relationships between TCI domains and antipsychotic treatment outcomes. Mean follow-up period was 9.5±1.8 years. SD was significantly anticorrelated with CPZ equivalents (Figure 2d, R^2^=−0.251, *P*=0.013, two-tailed). As expected, DUP was also associated with dose-years (Figure 2e, R^2^=0.193, *P*=0.032, two-tailed).

### Multidimensional personality profiles are associated with risk of schizophrenia

We estimated the associated risk of discrete multidimensional personality profiles by assessing differences in frequencies between risk groups according to the personality “cubes” schema. A summary of the distribution of risk groups (affected, unaffected relative or healthy control) within each personality profile is displayed in Table 3. The following paragraphs describe the impact of personality profiles on severity of parkinsonism and midbrain lesion size.

#### Temperament profiles

NHr (explosive/borderline) versus nhR (reliable). The ‘explosive’ personality type was significantly more frequent in subjects with schizophrenia than in relatives or controls, and significantly more frequent in relatives than in controls (*χ*^2^=12.946, *P*=0.002).

Nhr (adventurous) versus nHR (cautious). No difference was seen in the distribution of these profiles between risk groups.

NHR (sensitive) versus nhr (independent’). The ‘independent’ personality profile was significantly more frequent in subjects with schizophrenia than in relatives or controls, and more frequent in relatives than in controls (*χ*^2^=6.86, *P*=0.032).

nHr (methodical) versus NhR (passionate). The 'methodical' (nHr) profile was over-represented in schizophrenia (75%), whereas the ‘passionate’ profile was over-represented in healthy controls (72%), and unaffected relatives showed a similar frequency of both (*χ*^2^=10.40, *P*=0.006).

#### Character profiles

scT (schizotypal/disorganized’) versus Sct (organized’). The ‘schizotypal/disorganized’ (scT) type was over-represented in schizophrenia (91%), whereas the ‘organized’ (SCt) profile was over-represented in healthy controls (84%), and unaffected relatives had similar frequency of both (*χ*^2^=42.97, *P*<0.001).

sct (apathetic) versus SCT (creative). The ‘apathetic’ (sct) personality profile was significantly more frequent in subjects with schizophrenia, whereas the ‘creative’ profile was equally frequent in healthy controls and in unaffected relatives (*χ*^2^=18.46, *P*<0.001).

sCT (moody) versus Sct (bossy). There was a disproportion of the ‘moody’ profile (75%) in subjects with schizophrenia and the ‘bossy’ profile (77%) in healthy controls, while both profiles were equally frequent in unaffected relatives (*χ*^2^=7.09, *P*=0.029).

ScT (fanatical’) versus sCt (dependent’). No differences were seen for the personality subtypes between risk groups.

#### Resiliency profiles

hPS (resilient) versus Hps (fragile). The ‘fragile’ (Hps) profile was over-represented in schizophrenia (96%), and the ‘resilient’ (hPS) profile was over-represented in healthy controls (79%), while unaffected relatives were more frequently ‘fragile’ (58%) (*χ*^2^=36.35, *P*<0.001).

HpS (high-strung) versus hPs (happy-go-lucky). There was a disproportion of hPs personality profiles (80%) in the schizophrenia group, whereas 79% of healthy controls showed the complementary HpS personality profile (*χ*^2^=8.94, *P*=0.011). Similar proportions of each profile were found in unaffected relatives.

hps (laid-back) versus HPS (conscientious). The hps profile was over-represented in schizophrenia (92%), whereas each profile was equally likely in both unaffected relatives and healthy controls (*χ*^2^=17.90, P<0.001).

HPs (perfectionist) versus hpS (self-reliant). We observed a greater proportion of ‘perfectionist’ (HPs) profile (91%) in subjects with schizophrenia, whereas 71% of healthy controls were ‘self-reliant’ (hpS) (*χ*^2^=7.80, *P*=0.020). These two profiles were equally likely to be found in unaffected relatives.

## Discussion

The TCI has been extensively validated in subjects with schizophrenia and their unaffected relatives.^4,25,27,32–37^ More specifically, we previously showed that in the same population reported here subjects with untreated psychosis are able to complete the inventory, albeit on occasion they may require support to stay on task (they tend to drift off quite easily because of distracting internal processes). It is most worth pointing out, howerver, that subjects with untreated psychosis and their unaffected relatives deviate from healthy controls in predictable fashion, consistent across samples, indicating that the results are not artifactual or random. We evaluated a sample of subjects with chronic untreated schizophrenia at the time of initial assessment, as well as their unaffected first-degree relatives, and healthy matched controls. High HA, low RD, low SD, low CO and high ST was associated with greater risk of schizophrenia. Severity of parkinsonian motor impairment acted as a linear predictor of RD, CO, and SD after correcting for age and sex (Table 2 and Figures 2a–c). Unaffected relatives had higher ST and lower CO and SD than healthy controls, but lower than patients. These differences were documented in tests of individual traits, confirming prior work on risk of families of people medicated for schizophrenia.^23,24^

We extended prior findings by showing that specific multidimensional personality profiles, in which disorganization of emotion, abstract cognition, and motivation is prominent, are predicted by severity of dopaminergic dysfunction (measured by parkinsonian motor impairment), and found more frequently in subjects with chronic untreated schizophrenia and in their unaffected relatives than in healthy controls, consistent with an endophenotype (Table 3). Not surprisingly, some of the personality profiles associated with parkinsonism are also more frequent in subjects with schizophrenia than in their unaffected relatives, indicating a state-dependent marker (Table 3).

**Figure 3:**
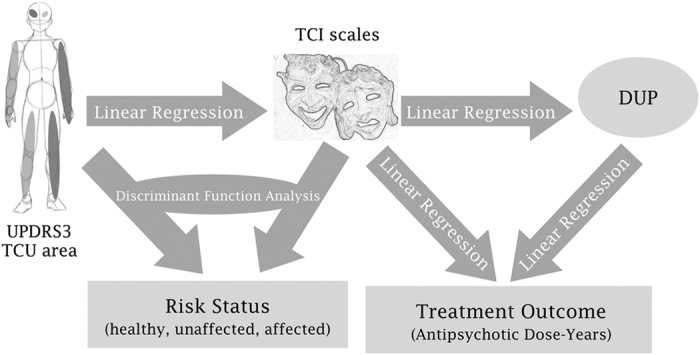
Hypothesis testing. Block arrows indicate the direction of prediction and the statistical method used to tested in each case.

Lastly, we found that SD predicted DUP and antipsychotic doses at follow-up (Figures 2d and e). SD was related to DUP and antipsychotic dose-years, such that lower SD predicted longer DUP and higher antipsychotic dosages at follow-up (Figures 2f and Figure 3).

In the next paragraphs we will consider individual traits first, because prior data is limited to such observations. Then we will discuss the associations of multidimensional personality profiles with specific disorganized and negative features underlying risk some forms of the schizophrenias.

Lastly, we will discuss the role of dopamine deficits expressed by parkinsonian motor impairment and hyperechogenicity of the substantia nigra as a pathogenic mechanism mediating the relation of personality profiles to clinical outcomes in schizophrenia.

### Parkinsonism and individual personality traits in schizophrenia

Our findings (high HA, ST and low RD, CO, SD) approximate those of the only available meta-analysis.^35^ Prior studies, however, included subjects either chronically treated or during the first episode of illness. Our study is the first to report on subjects with chronic untreated symptoms at the time of assessment, likely reflecting personality traits intrinsic to the disease process. In unaffected relatives, we found high ST with low CO and SD, similar to what is seen in schizotypal personality disorder.^40^ Since patients and unaffected relatives share similar genetic contributions and, at least in part, similar environmental challenges, our design cannot distinguish the influence of common environmental factors on temperament and character from that of genetic liability factors.

HA has been proposed as a core etiopathologic feature of schizophrenia.^24,37,42^ We found that severity of parkinsonism acted as a linear predictor of HA, but on average the trait did not differ between unaffected relatives and healthy controls. We also found that RD, CO and SD are predicted by biological markers of dopaminergic function (parkinsonism for all three, and midbrain hyperechogenicity for RD), suggesting that they are related to the underlying pathophysiology. For CO and SD, unaffected relatives express an intermediate phenotype between patients and controls.

Interestingly, RD appears to affect the expression of negative symptoms.^38,41^ Neuroimaging studies of RD support a role for basal ganglia in its expression^29–31^ and as a putative disease mechanism.^29,43^ Conversely, high levels of CO and SD have been linked to positive outcomes suggesting a possible role in resilience.^23,24,44,45^

Not surprisingly, unaffected relatives who were misclassified as having schizophrenia in our discriminant analysis, showed lower RD, CO, SD than those correctly classified or those misclassified as healthy controls. These findings are similar to studies in ultra high-risk subjects.^41^

The same misclassified unaffected relatives also showed greater parkinsonism than other unaffected relatives. Lower CO and SD have been thought to reflect risk for psychosis.^23,24,45^ In ultra high-risk samples lower CO has been shown to predict transition to manifest psychosis.^46^ Relatives misclassified as having schizophrenia also had lower RD, possibly representing a tendency for social withdrawal, as RD measures social attachment and dependence. In line with this reasoning, studies in prodromal psychosis have found greater social anhedonia and withdrawal as predictors of conversion to psychosis,^47,48^ and others suggested that RD is associated to genetic vulnerability.^41^

Even if high-risk individuals never transition to manifest psychosis, relatives with low CO, SD, and RD may experience subclinical phenomena that can contribute to significant social dysfunction and a reduced quality of life.^41,49^ In summary, low RD, CO, SD, when combined with clinically significant parkinsonism, may represent markers of genetic risk for psychosis.

Lower SD predicted longer DUP in subjects in a sample where the mean DUP was >5 years. To the best of our knowledge ours is the first report of a personality domain as a linear predictor of DUP in schizophrenia, as the only previous data comes from subjects with first-episode psychosis^50^ (and therefore a less stable diagnoses and shorter DUP).^51^ This finding has clinical implications as a personality domain intrinsic to the disease process is related to longer DUP, likely leading to worse clinical outcomes.^52^ Also, ours is the first study relating baseline personality measures obtained before the initiation of treatment, to prediction of treatment outcomes. We found that lower SD before the initiation of treatment predicts greater antipsychotic dosing on follow-up, which can be viewed as a proxy for treatment refractoriness. Longer DUP was also related to higher dose-years. Taken together, a possible interpretation of the data would be that SD contributes to personality profiles such that these individuals suffer from prolonged social isolation and longer DUP, resulting in more severe illness course and poorer outcomes.

### Parkinsonism and multidimensional personality profiles in schizophrenia

However, when multidimensional personality profiles were considered, we found that in addition to *schizotypal* personality (low SD, CO, and high ST), others subjects with chronic schizophrenia may express *explosive* (*borderline*) temperament (high NS, HA and low RD) and 'methodical (obsessional)' temperament (low NS, high HA, and low RD). In our prior work we reported averages for individual traits;^4^ now we extended our analyses to individual multidimensional profiles and tested their association with schizophrenia and with parkinsonism. Our findings demonstrate that individuals with 'explosive' or 'methodical' temperament profiles, 'disorganized' (schizotypal) character profile, and with plasticity profiles described as ambivalent in motivation to act, have more severe parkinsonian motor signs (see Table 3) and are at greater risk of schizophrenia (see Table 3).

Individuals with ‘explosive*’* (high NS, HA, and low RD) and ‘*methodical’* (low NS, high HA, and low RD) temperament profiles; ‘schizotypal/disorganized’ (low SD, CO and high ST) character profiles; and ‘fragile’ (high HA and low PS, SD) and ‘laid-back’ (low HA, PS, SD) resiliency profiles had greater parkinsonian motor signs and risk of schizophrenia. Only subjects with the ‘borderline’ temperament profile had significantly larger echogenic area of the right SN on average. It is worth noting that the previous analysis modeled both personality profiles with clinical and imaging markers of parkinsonism, therefore the associations with increased risk are in light of clinical parkinsonism.

We further assessed whether these personality profiles associated with parkinsonism individually predicted risk of schizophrenia. Interestingly, ‘borderline’ (OR: 4.3, CI: 1.3–14.1, *P*=0.01), ‘methodical’ (OR: 4.3, CI: 1.6–11.8, *P*=0.003), and ‘schizotypal’ (OR: 9.4, CI: 3.5–24.9, *P*<0.001) were associated with increased risk of schizophrenia, whereas the ‘fragile’ and ‘laid-back’ profiles were not associated with schizophrenia in the absence of parkinsonism.

Studies of the premorbid personality of patients with Parkinson disease without a history of psychosis have previously shown an association with low NS and high HA.^20,53^ Low NS is associated with decreased D2 availability in the insular salience network and a deficit in the mesolimbic branch of ascending dopamine transmission in the left hemisphere,^54^ whereas increased harm avoidance is associated with greater dopamine loss in the right striatum^53^ and increased connectivity of the right anterior insula with the anterior cingulate and dorsolateral prefrontal cortex.^55^ Low NS also is strongly related to reduced dopamine synthesis in the ventral striatum and increased midbrain dopamine autoreceptor availability.^56^ These individual differences in dopaminergic neurotransmission underlie vulnerability to parkinsonism and to disturbance of the prefrontal-basal ganglia-prefrotal loop that may underlie 'disorganized (hebephrenic)' personality types and negative symptoms, including avolition, blunting of affect, and ambivalent decision making,^57^ which is prominent in particular subtypes of schizophrenia.^58^ Our findings specifically show that specific personality profiles characterized by 'asociality' (high HA, low RD), 'ambivalence' (low PS, SD), and 'disorganized thinking' (low SD, CO, high ST) are associated with more severe parkinsonian motor symptoms.

These findings provide a well-documented mechanism for the relationship of personality to parkinsonism and the pathogenic role of both on some forms of the schizophrenias. Furthermore, our findings suggest the possibility that specific subsets of the subjects with chronic untreated schizophrenia ascertained in the Andes mountains may share pathogenetic mechanisms. For instance, the specific disorganized subtype of schizophrenia we observed in this sample, was assocaited with several genes that act in concert with an abnormal GOLGA1 gene.^58^ Indeed, we previously showed that in the Kechwa sample parkinsonism has a significant association with disorganized and negative symptoms^7^ and with specific genome-wide genotypes.^59^ Further studies of the phenotypic and genotypic profiles of this subgroup are warranted to test this hypothesis.

### Conclusions

Our study is the first to relate personality profiles to a discrete well-defined neurological deficit with a known etiopathologic mechanism, as parkinsonism predicts core features of the personality structure of at least a subtype of the schizophrenias. Further, our study extends these observations to unaffected relatives suggesting that a personality profile can act as an endophenotype of schizophrenia and is therefore a marker of genetic risk. Lastly, we found that both parkinsonism and personality traits at initial assessment are predictive of clinical outcomes and course of illness up to ten years later. An important limitation to our study is the loss of sample size on follow-up, even though retention rate was quite satisfactory for a study lasting a decade. Lastly, the fact that our data originate from subjects from a specific ethnic group in South America may limit its generalizability. Yet, the fact that the direction and profile of temperament and character deviance in our sample is very similar to that reported in samples in other countries and cultures—including a meta-analysis—is very encouraging and supportive of a generalized pathophysiological process, due to the disease and not to the ethnicity or culture of the sample.

Nonetheless, we believe our data strongly suggest that baseline personality profiles influence the expression and course of schizophrenia, therefore warranting further investigation.

## Materials and methods

### Population and sampling

The recruitment and ascertainment methods of this study were recently published.^6,7^ The study was carried out in the Province of Jujuy, Argentina (population 650 000). Briefly, primary care health agents are in direct contact with the entire population of the province, and in rural areas visit every household at least twice a year. When a health agent detects a possible case of severe mental illness, a notification is forwarded to the investigators using the epidemiological surveillance system of the province. This system was originally intended for detection/notification/surveillance of transmissible diseases (such as yellow fever, dengue fever, tuberculosis, etc). We trained health agents to detect, refer and follow-up cases of severe mental illness.^7^ Once a case was detected, the mobile research team contacted them to carry out an evaluation, which was carried out at the subject's closest health unit. At the outset of the assessment, participation in research was offered to the subject, his/her unaffected relatives if available, and to a neighbor matching the subject in age and sex identified by the health agent.^6,7^ If participation in research was not accepted, the assessment and treatment proceeded through the Mental Health system of the province, which provides universal care. If research participation was accepted, all assessments were carried out blindly by separate members of the team after written informed consent was completed and included diagnostic ascertainment, neurological, and neuropsychological examinations.^5–7^ Diagnostic ascertainment was carried out using the World Health Organization's Schedules for Clinic Diagnosis in Neuropsychiatry (SCAN).^60^ Duration of untreated psychosis (DUP) was also assessed using SCAN with direct questioning of the subject, collateral information from family members and any alternate sources of information, such as health or school records. Subjects who met DSM-IV-TR criteria for schizophrenia and were never exposed to antipsychotic medications were included in the study. We also recruited at least one sibling or parent to participate, and a healthy volunteer from the same neighborhood matched by age, sex, and education to the index subject. Relatives and healthy controls were also interviewed with SCAN. Additional inclusion criteria were: (i) age between 18 and 65; and (ii) no neurological or substance abuse comorbidity. All research procedures, consents, and forms were independently approved by the Internal Review Board of the Morsani College of Medicine at University of South Florida, and by the Ethics Committee of the Ministry of Public Health of the Province of Jujuy, Argentina.

### Temperament and character inventory

The Temperament and Character Inventory (TCI) is a 240 item self-report assessment of personality dimensions.^61^ TCI measures temperament (i.e., novelty seeking (NS), harm avoidance (HA), RD, persistance (PS)) and character (i.e., SD, cooperativeness (CO), ST). Temperament refers to relatively automatic responses to perceptual and emotional stimuli that are part of the individual's innate behavioral repertoire, whereas character domains reflect learned differences in the attribution of salience and self-concepts. Specifically, NS is an expression of an individual’s likeliness to seek new experiences and novel rewards; HA reflects a propensity for fatigability and the avoidance of punishment; RD resonates a tendency for social attachment and dependence; PS taps in to an individual’s conscientious and compulsive qualities; SD measures one’s adaptability to environmental consequences; CO typifies a sense of agreeableness and the ability to get along with people; and ST reflects the individual’s integration as a part of the greater universe. To analyze the impact of multidimensional profiles on risk for schizophrenia, we first partitioned the population according each subject's position above (capital letter) or below (small-caps) the median for each scale (HA -H or h-, NS -N or n-, RD -R or r-, P -P or p-, SD -S or s-, C -C or c-, and ST -T or t-). We then formed personality profiles according to the three personality “cubes” – temperament, character, and resilience, respectively – by assigning each case (regardless of diagnostic status) to one of the following dichotomous pairs (see Table 3). It should be noted that the labels for each profile are approximate descriptions of its salient features and should not be taken as comprehensive descriptions.

### Motor assessment

A detailed description was published elsewhere.^5^ Parkinsonism was scored blindly using Unified Parkinson's Disease Rating Scale (UPDRS3) on videotaped exams by independent certified raters. The scale rates speech, facial expression, tremor, bradykinesia, akinesia, rigidity, posture and postural instability, and gait. Since rigidity could not be directly assessed on the videotapes it was not scored. The minimum clinically significant change in UPDRS-3 scores has been shown to be >6.^62^

### Transcranial ultrasound

Transcranial ultrasound was carried out as previously described.^5^ Briefly, we employed a 2.5 MHz transducer (Micromaxx, Sonosite Inc, Bothell, WA, USA) to examine the brainstem through a pre-auricular acoustic bone window (penetration depth=16 cm, dynamic rane=45 dB) by an expert sonographist. The substantia nigra was identified within the butterfly-shaped structure of the brainstem, scanning from each temporal bone window. Unbiased quantification of the echogenic area was carried out *post hoc* on saved images by two different evaluators blind to subject's condition. Sonographic measurements proved adequately reproducible.

### Long-term clinical outcomes

We conducted a follow-up of all affected subjects enrolled in our study.^7^ For this analysis we included treatment outcome data from individuals who completed the TCI assessment at ascertainment and had adequate medical records. Cumulative neuroleptic exposure was computed in dose-years,^63^ after conversion to chlorpromazine equivalents using published tables.^64,65^ A dose-year is defined as the product of the chlorpromazine equivalents and the time on each dose expressed in years.

### Statistical analysis

All variables with significant skew deviation or kurtosis were normalized using log transformations where appropriate. Group differences for demographic variables were assessed by one-way ANOVA for diagnostic categories. MANOVA was used to assess the effect of risk status (healthy, unaffected relative, affected subject) on individual TCI scales with age, sex, and education as covariates, followed by *post hoc* Bonferroni (IBM SPSS, Armonk, NY, USA). Figure 3 describes the sequence of analysis performed and the hypothesis tested. To assess the influence of dopaminergic function on personality domains we derived linear regressions for individual TCI scales using UPDRS3 and ultrasound as predictors, with age and sex as covariates. We used a linear discriminant analysis to determine if TCI scales, UPDRS3, and ultrasound, could predict risk status (i.e., schizophrenia, unaffected relative, control). A *post hoc* MANOVA analysis of unaffected relatives who were misclassified as ‘schizophrenia’ against all other unaffected relatives in the sample was carried out to assess for subtle clinical manifestations that could be used to screen for vulnerability to psychosis. A correlational analysis was conducted between DUP and TCI domains and between DUP and TCI domains on treatments outcomes (i.e., chloropromazine equivalents and dose-years). To examine the relationship of discrete configurations of personality profiles with risk of schizophrenia, we estimated the proportion of cases, unaffected relatives and affected subjects for each opposing pair of personality profiles using Chi square distributions, and we calculated the odds ratios for risk profiles associated with schizophrenia. All calculations were performed on the statistical package SPSS version 22 (IBM Corp, Armonk, NY, USA).

## Figures and Tables

**Table 1:** Summary of demographic, clinical, and personality variables

	*Schizophrenia*		*Relatives*		*Controls*		*F*	P
	*Mean*	*s.e.m.*	*Mean*	*s.e.m.*	*Mean*	*s.e.m.*		
Age	28.0	1.4	40.4	1.5	30.4	1.1	22.4	<0.001
Sex	37M/24F	—	36M/64F	—	35M/33F	—	10.0	0.007
Education	10.0	0.3	9.9	0.4	11.1	0.4	2.9	0.058
								
*TCI domains—temperament*								
Novelty seeking	96.0	1.8	93.3	1.4	95.4	0.9	1.2	0.309
Harm avoidance	112..8	2.5	105.7	1.5	102.0	1.7	3.5	0.010
Reward dependence	86.6	1.9	100.4	1.6	103.3	1.9	11.2	<0.001
Persistence	103.8	2.7	114.7	2.0	113.1	2.4	2.1	0.089
								
*TCI domains—character*								
Self-directedness	115.0	2.7	139.7	2.2	151.8	2.5	20.0	<0.001
Cooperativeness	119.2	2.3	131.1	1.6	137. 4	2.0	8.8	<0.001
Self-transcendence	80.8	2.4	76.9	1.8	68.6	2.0	3.4	0.011
UPDRS-3	16.2	1.0	6.9	0.6	4.6	0.6	31.1	<0.001
Substantia nigra echogenicity left (cm^2^)	0.21	0.03	0.19	0.02	0.2	0.03	1.9	0.111
Substantia nigra echogenicity right (cm^2^)	0.25	0.03	0.16	0.02	0.14	0.03	7.3	<0.001

Abbreviation: TCI, Temperament and Character Inventory.

**Table 2:** Summary of regression analysis

	*Parkinsonism*		*Right SN echogenicity*		*Corrected for multiple comparison*		
	*β*	p	*β*	P	*R2*	*F*	P
Harm avoidance	0.21	0.007	0.11	0.194	0.063	2.9	0.025
Reward dependence	−0.39	<0.001	−0.17	0.019	0.265	15.3	<0.001
Persistence	−0.23	0.003	−0.01	0.887	0.057	2.6	0.039
Self-directedness	−0.36	<0.001	−0.07	0.346	0.183	9.5	<0.001
Cooperativeness	−0.26	0.001	−0.12	0.137	0.108	5.2	0.001
Self-trancendensce	0.13	0.092	−0.004	0.966	0.025	1.1	0.368

Linear regressions were used to assess the role of parkinsonism and it’s biomarker, the area of SN echogenicity on personality domains that demonstrated group-level differences. Age and sex were used as covariates.

**Table 3:** *χ*^2^ distributions of personality profiles by risk status

*Contrast (n)*	*Schizophrenia*	*Unaffected*	*Healthy*	*χ*^2^	P
*Temperament*					
Explosive *'NHr'* (21) versus reliable *'nhR'* (26)	86.7 vs. 13.3	43.3 vs. 56.7	26.7 vs. 73.3	12.946	0.002
Adventurous *'Nhr'* (23) versus cautious *'nHR'* (15)	64.3 vs. 36.7	32.5 vs. 67.5	60 vs. 40	3.078	0.218
Independent *'nhr'* (18) versus sensitive *'NHR'* (18)	87.5 vs. 12.5	44.4 vs. 55.6	36.4 vs. 63.6	6.859	0.032
Methodical *'nHr'* (27) versus passionate *'nHR'* (27)	75 vs. 25	46.4 vs. 53.6	28.6 vs. 71.4	10.393	0.006
					
*Character*					
Organized *'SCt'* (42) versus schizotypal *'scT'* (39)	9.4 vs. 90.6	47.2 vs. 52.8	83.8 vs. 16.2	42.968	<0.001
Apathetic *'sct'* (19) versus creative *'SCT'* (24)	92.9 vs. 7.1	33.3 vs. 66.7	25 vs. 75	18.455	<0.001
Bossy *'Sct'* (19) versus moody *'sCT'* (17)	25 vs. 75	53.8 vs. 46.2	76.9 vs. 23.1	7.093	0.029
Dependent *'sCt'* (9) versus fanatical *'ScT'* (6)	100 vs. 0	37.5 vs. 62.5	66.7 vs. 33.3	4.812	0.090
					
*Resiliency*					
Fragile *'Hps'* (31) versus resilient *'hPS'* (36)	95.8 vs. 4.2	42.4 vs. 57.6	20.7 vs. 79.3	36.351	<0.001
Happy-go-Lucky *'hPs'* (16) versus high-strung *'HpS'(12)*	80 vs. 20	55.6 vs. 44.4	21.4 vs. 78.6	8.937	0.011
Conscientious *'HPS'* (15) versus laid-back *'hps'* (15)	7.7 vs. 92.3	78.6 vs. 21.4	75 vs. 25	17.895	<0.001
Perfectionist *'HPs'* (23) versus self-reliant *'hpS'* (27)	78.6 vs. 21.4	51.4 vs. 48.6	29.4 vs. 70.6	7.798	0.020
