# Supplementary material for: Parkinsonian motor impairment predicts personality domains related to genetic risk and treatment outcomes in schizophrenia
Source: NPJ Schizophr. 2017 Jan 11;3:16036–. doi: 10.1038/npjschz.2016.36 (PMC5226082; doi:10.1038/npjschz.2016.36)
Supplement: Supplementary Table 1 [file npjschz201636-s1.doc]

Supplementary Table 1. Temperament, Character and Resilience Profiles Derived from TCI
